# Arterial lactate and enteral feeding intolerance within the vasopressor gray zone: a retrospective cohort study

**DOI:** 10.3389/fnut.2026.1864125

**Published:** 2026-07-24

**Authors:** Qinqin Shu, Min Tang, Qi Si, Jiuchen Xu, Yan Li

**Affiliations:** Department of Emergency Medicine, Shanghai Fourth People’s Hospital, School of Medicine, Tongji University, Shanghai, China

**Keywords:** arterial lactate, critical care, enteral nutrition, feeding intolerance, intensive care unit, vasopressors

## Abstract

**Background:**

Enteral feeding tolerance during vasopressor therapy remains uncertain, particularly within the moderate-dose gray zone. This preregistered analysis tested whether pre-feeding arterial lactate modifies the dose–response relationship between norepinephrine-equivalent dose and enteral feeding intolerance (vomiting).

**Methods:**

Using the same MIMIC-IV cohort (*n* = 8,055), we examined whether baseline arterial lactate (most recent value within 24 h before enteral nutrition initiation) modifies the norepinephrine-equivalent dose–vomiting association. Effect modification was assessed via multiplicative interaction in multivariable logistic regression and stratified restricted cubic spline analyses, with lactate dichotomized at 2 mmol/L. A joint risk surface was constructed using generalized additive modeling.

**Results:**

Among 5,542 patients with available lactate (68.8% of the parent cohort), 278 (5.0%) developed vomiting. The global test for multiplicative interaction was not significant (*p* = 0.27). Exploratory stratified analysis showed a localized signal at doses of 0.3–0.5 μg/kg/min: vomiting was 9.8% among patients with lactate >2 mmol/L versus 4.9% among those with lactate ≤2 mmol/L. Separately, in the dichotomized joint-exposure model, norepinephrine-equivalent dose ≥0.3 μg/kg/min plus lactate >2 mmol/L was associated with an adjusted OR of 2.02 (95% CI 1.33–3.07; *p* = 0.001) versus dose <0.3 μg/kg/min plus lactate ≤2 mmol/L. Dose–response curves showed steeper risk escalation in the elevated-lactate stratum, though confidence bands overlapped throughout; this divergence was directionally consistent across alternative lactate thresholds (1.5 and 4.0 mmol/L) and exposure windows (6-h, 12-h).

**Conclusion:**

At moderate vasopressor doses, elevated arterial lactate may identify a subgroup with a higher observed vomiting rate—an exploratory signal not fully captured by vasopressor dose alone. If confirmed prospectively, this pattern could inform a lactate-stratified approach to enteral feeding advancement: at norepinephrine-equivalent 0.3–0.5 μg/kg/min, lactate >2 mmol/L might support slower advancement with closer monitoring rather than standard escalation. Given that feeding intolerance is a key driver of enteral nutrition interruption, these findings have potential downstream implications for nutritional adequacy and clinical outcomes. Because the global interaction was not statistically significant, these findings are hypothesis-generating and require prospective validation.

## Introduction

Enteral nutrition (EN) is the preferred route of nutritional support in critically ill patients, yet its safe initiation during vasopressor therapy remains debated (1, 2). Balancing the benefits of early EN against gastrointestinal complications when splanchnic perfusion is compromised remains a key challenge (3, 4). In our companion study (5), we identified a non-linear dose–response between norepinephrine equivalent (NEQ) dose and overt EN intolerance (vomiting) in 8,055 MIMIC-IV patients, with a risk inflection around 0.3 μg/kg/min. That analysis also showed that composite feeding intolerance (FI) definitions incorporating lactate inflated apparent risk, highlighting circularity between NEQ and lactate as co-markers of shock severity.

However, that analysis examined lactate only as an outcome component, not as a potential effect modifier. Arterial lactate may capture hemodynamic compromise not reflected by vasopressor dose alone (6–8), suggesting that the “safe” vasopressor threshold for EN initiation may depend on concurrent perfusion status. The splanchnic circulation is particularly vulnerable to hypoperfusion (9, 10); when exogenous vasopressors are superimposed on global hypoperfusion (indexed by elevated lactate), mesenteric blood flow reduction may be associated with a disproportionate increase in FI risk (11, 12). This preregistered analysis tested whether baseline arterial lactate modifies the NEQ–FI dose–response curve, with secondary aims of constructing a joint risk surface and evaluating incremental discrimination. The protocol was prospectively registered on the Open Science Framework^1^.

## Materials and methods

### Study design, data source, and ethical approval

This preregistered retrospective cohort study used the same MIMIC-IV (v2.0) cohort as the parent study (5, 13). This study followed the STROBE guidelines (14).

### Study population

The study population was identical to that previously described (n = 8,055); no additional exclusions were applied.

Eligibility criteria (restated for self-containment). Adult ICU patients (≥18 years) in MIMIC-IV v2.0 were included if they received at least one vasopressor infusion and were started on enteral nutrition during the ICU stay, with enteral nutrition initiated after the first vasopressor. Patients without any charted vasopressor infusion, without an identifiable enteral-nutrition start, or younger than 18 years were excluded. For the present interaction analysis, the analytic sample additionally required a baseline arterial lactate value within the 24 h before enteral-nutrition initiation; patients without an eligible lactate value were excluded from interaction models, yielding 5,542 patients, and we compared the characteristics of patients with and without available lactate to assess generalizability.

Ethical approval (self-contained). MIMIC-IV is a publicly available, de-identified database whose original collection was approved by the institutional review boards of Beth Israel Deaconess Medical Center and the Massachusetts Institute of Technology (13). Because the database is fully de-identified and publicly available, this secondary analysis required no additional institutional review board approval, and the requirement for individual informed consent was waived, in accordance with local legislation and institutional requirements. The authors are credentialed PhysioNet users who completed the required human-subjects research training and accessed the data under the PhysioNet data-use agreement.

### Exposure variables

The primary exposure was maximum NEQ dose in the 24 h preceding EN initiation (5, 15). NEQ was modeled using restricted cubic splines (RCS) with 4 knots.

Vasopressor extraction and norepinephrine-equivalent (NEQ) calculation. Vasopressor infusions were extracted from the MIMIC-IV icu.inputevents table for five agents identified by itemid: norepinephrine, epinephrine, phenylephrine, dopamine, and vasopressin. Dobutamine, although present in the infusion records, was excluded as an inotrope rather than a vasopressor. Infusion rates were recorded in weight-indexed units (μg/kg/min) using the charted patient weight; vasopressin (units/min) was the only non-weight-indexed agent. Each agent was converted to norepinephrine equivalents following Goradia et al. (15) using the formula NEQ (μg/kg/min) = norepinephrine + epinephrine + phenylephrine/10 + dopamine/100 + vasopressin (units/min) × 2.5. Concurrent infusions of different agents were summed at each charted time interval to yield a single instantaneous NEQ rate, and the maximum NEQ rate within the 24 h preceding enteral-nutrition initiation was used as the exposure. Implausible values were filtered (non-positive infusion rates set to missing), and infusion intervals were defined by charted start and stop times.

The effect modifier was baseline arterial lactate (most recent value within 24 h before EN initiation), dichotomized at 2 mmol/L (16, 17). Secondary analyses used continuous lactate and alternative thresholds (1.5 and 4 mmol/L).

Lactate was obtained from the MIMIC-IV hosp.labevents table (laboratory itemid for arterial/blood lactate), restricted to physiologically plausible values (0–25 mmol/L). For each patient we used the single most recent lactate value charted within the 24 h before enteral-nutrition initiation as the baseline value; patients without an eligible value in this window were excluded from the interaction models.

### Primary outcome

The primary outcome was clinically documented vomiting during EN, selected for gastrointestinal specificity and to avoid circularity between lactate-as-modifier and lactate-as-outcome (5).

Operational definition of vomiting. Vomiting was ascertained from structured nursing documentation in the MIMIC-IV chartevents table, using the gastrointestinal-assessment itemids together with value text matching the pattern “vomit” or “emesis”; International Classification of Diseases codes were not used, as they under-capture symptomatic in-ICU events. A patient met the outcome if at least one vomiting episode was documented after enteral-nutrition initiation within the observation window; multiple charted entries for the same patient were collapsed to a single binary patient-level event, and negated entries (e.g., “no vomiting”) were excluded by the matching logic. Enteral nutrition was identified using a prespecified, locked whitelist of 75 standalone enteral-formula itemids, with protein additives and all parenteral-nutrition products excluded, so that the outcome was assessed only against genuine enteral-nutrition exposure.

### Covariates

Covariates were age, sex, non-cardiovascular SOFA score, time to EN, and ICU type, identical to those previously described (5).

### Statistical analysis

*Interaction analysis.* The multiplicative interaction between RCS-modeled NEQ and dichotomized lactate was tested in a fully adjusted logistic regression using a likelihood ratio test. Additive-scale interaction was quantified using relative excess risk due to interaction (RERI), attributable proportion (AP), and synergy index (SI) with dichotomized NEQ (≥0.3 vs. < 0.3 μg/kg/min) and lactate (>2 vs. ≤ 2 mmol/L) (18, 19).

*Stratified dose–response analysis.* Stratified RCS dose–response curves were generated for lactate-normal (≤2 mmol/L) and lactate-elevated (>2 mmol/L) strata as a prespecified analysis.

*Joint risk surface.* A joint risk surface was constructed using a generalized additive model (GAM) with a tensor product smooth of NEQ and lactate.

*Sensitivity analyses. Seven prespecified sensitivity analyses tested robustness, including alternative lactate thresholds (1.5, 4.0 mmol/L), continuous lactate interaction, 6-h and 12-h exposure windows, adjustment for mechanical ventilation, and restriction to medical ICU patients.*

*Discrimination analysis.* Incremental discrimination was evaluated by comparing AUC between the base model and the lactate-expanded model using the DeLong test (20).

Analyses used R version 4.3.0. A two-sided *p* < 0.05 was considered significant. Because this was a preregistered secondary analysis of all eligible patients, no formal *a priori* sample size calculation was performed; with 278 events and 7 predictor terms (including the interaction), the events-per-variable ratio exceeded 35, supporting stable model estimation. Given the prespecified hypothesis and fixed cohort size, the study was powered to detect clinically meaningful main effects, while interaction analyses were interpreted as exploratory signals.

## Results

### Baseline lactate distribution

Of 8,055 patients, 5,542 (68.8%) had baseline lactate available and comprised the analytic sample: 4,353 (78.5%) with lactate ≤2 mmol/L and 1,189 (21.5%) with lactate >2 mmol/L. Vomiting occurred in 278 (5.0%) patients. Patients with elevated lactate had higher illness severity, higher mean NEQ, and greater representation in higher NEQ categories (Table 1).

**Table 1:** Baseline characteristics stratified by lactate group (≤2 vs. >2 mmol/L) (*n* = 5,542).

Characteristic	Normal lactate (≤2 mmol/L), *n* = 4,353	Elevated lactate (>2 mmol/L), *n* = 1,189	*P*
Age, mean (SD), years	64.97 (15.41)	64.10 (15.49)	0.084
Sex, male, *n* (%)	2,575 (59.2)	677 (56.9)	0.14
Non-CV SOFA, mean (SD)	0.70 (1.61)	1.25 (2.31)	<0.001
ICU length of stay, mean (SD), days	13.32 (10.83)	12.54 (10.08)	0.027
Time to EN, mean (SD), hours	63.58 (55.53)	58.23 (55.26)	0.003
Max NEQ 24 h, mean (SD), μg/kg/min	0.18 (1.12)	0.30 (1.44)	0.003
NEQ < 0.1, *n* (%)	2,347 (53.9)	438 (36.8)	<0.001
NEQ 0.1–0.2, *n* (%)	1,017 (23.4)	248 (20.9)	
NEQ 0.2–0.3, *n* (%)	448 (10.3)	174 (14.6)	
NEQ 0.3–0.5, *n* (%)	347 (8.0)	204 (17.2)	
NEQ ≥ 0.5, *n* (%)	194 (4.5)	125 (10.5)	
Medical ICU, *n* (%)	1,952 (44.8)	661 (55.6)	<0.001
Surgical ICU, *n* (%)	1,262 (29.0)	321 (27.0)	
Cardiac ICU, *n* (%)	942 (21.6)	173 (14.6)	
Other ICU, *n* (%)	197 (4.5)	34 (2.9)	
Mechanical ventilation, *n* (%)	3,718 (85.4)	1,002 (84.3)	0.38
Vomiting (outcome), *n* (%)	207 (4.8)	71 (6.0)	0.11

NEQ, norepinephrine-equivalent dose; SOFA, Sequential Organ Failure Assessment; CV, cardiovascular; ICU, intensive care unit; EN, enteral nutrition; SD, standard deviation. *p* values from two-sample *t* tests (continuous variables) or *χ*^2^ tests (categorical variables).

### Interaction between NEQ and lactate

The likelihood ratio test for the multiplicative NEQ × lactate interaction was not significant (*χ*^2^ = 5.17, df = 4, *p* = 0.27). Stratum-specific ORs for NEQ 0 → 0.5 were numerically larger in the elevated-lactate group (1.53 vs. 1.08), but confidence intervals overlapped. Cross-tabulation (Table 2; Supplementary Figure S1) revealed the largest divergence at NEQ 0.3–0.5, where vomiting was 9.8% (20/204) with elevated lactate versus 4.9% (17/347) with normal lactate.

**Table 2:** Vomiting rates by NEQ dose category and lactate stratum (*n* = 5,542).

NEQ category (μg/kg/min)	Normal lactate (≤2 mmol/L): *n*; vomiting *n* (%)	Elevated lactate (>2 mmol/L): *n*; vomiting *n* (%)
<0.1	2,347; 112 (4.8%)	438; 25 (5.7%)
0.1–0.2	1,017; 44 (4.3%)	248; 9 (3.6%)
0.2–0.3	448; 19 (4.2%)	174; 8 (4.6%)
0.3–0.5	347; 17 (4.9%)	204; 20 (9.8%)
≥0.5	194; 15 (7.7%)	125; 9 (7.2%)
Total	4,353; 207 (4.8%)	1,189; 71 (6.0%)

Cells show stratum *n*; vomiting events *n* (%). NEQ, norepinephrine-equivalent dose.

### Stratified dose–response curves

**Figure 1:**
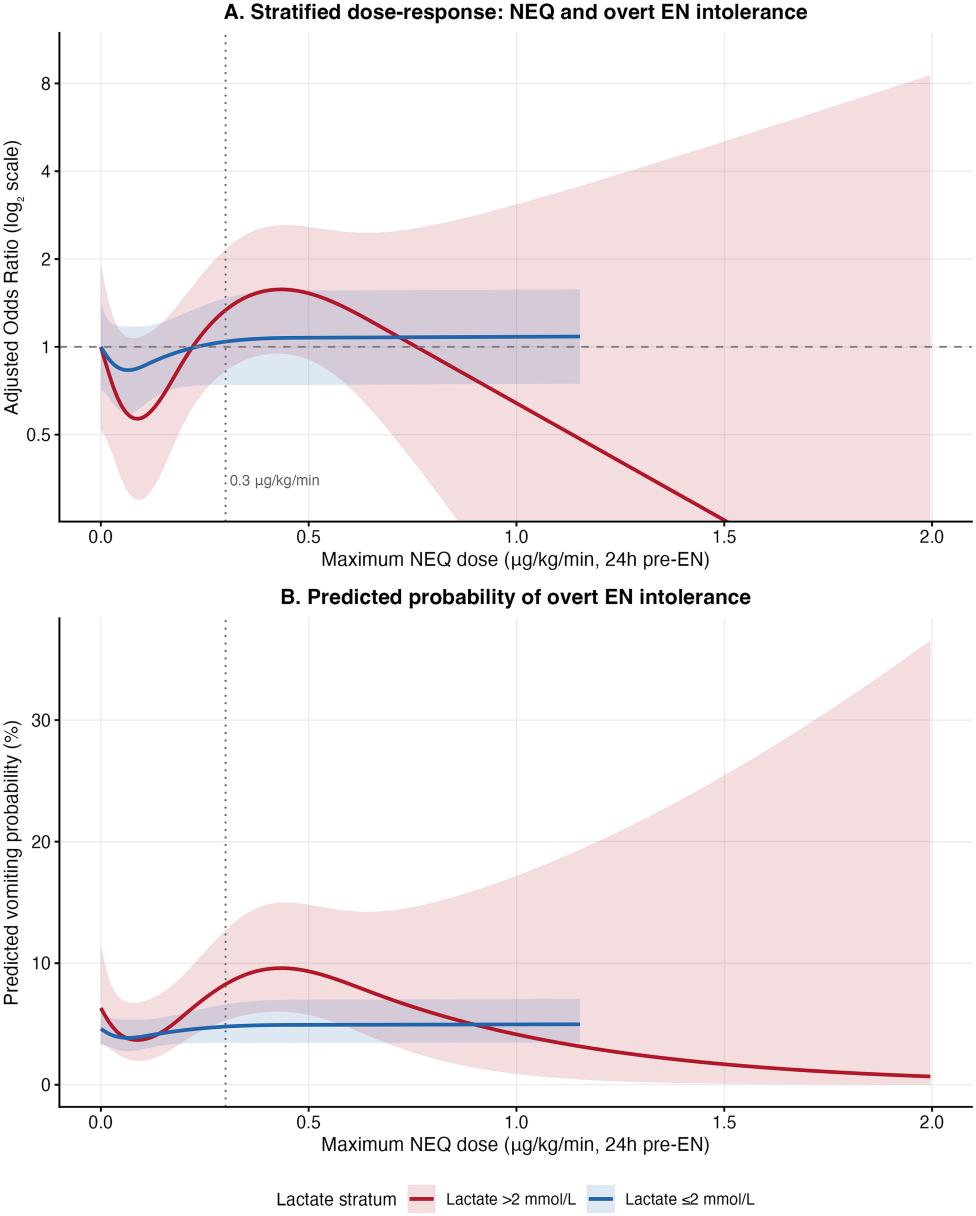
Stratified restricted cubic spline dose–response curves for vomiting risk by lactate stratum. Panel **(A)**: adjusted odds ratios. Panel **(B)**: predicted probabilities. Shaded regions, 95% CIs. Blue, lactate ≤2; red, >2 mmol/L.

Stratified RCS curves (Figure 1) showed similar baseline risk at NEQ = 0 (approximately 4.5–5.5%) but progressive divergence at higher doses. The elevated-lactate stratum exhibited steeper risk escalation, particularly above NEQ 0.3, though confidence bands overlapped throughout, consistent with the non-significant global test.

### Joint NEQ × lactate risk surface

**Figure 2:**
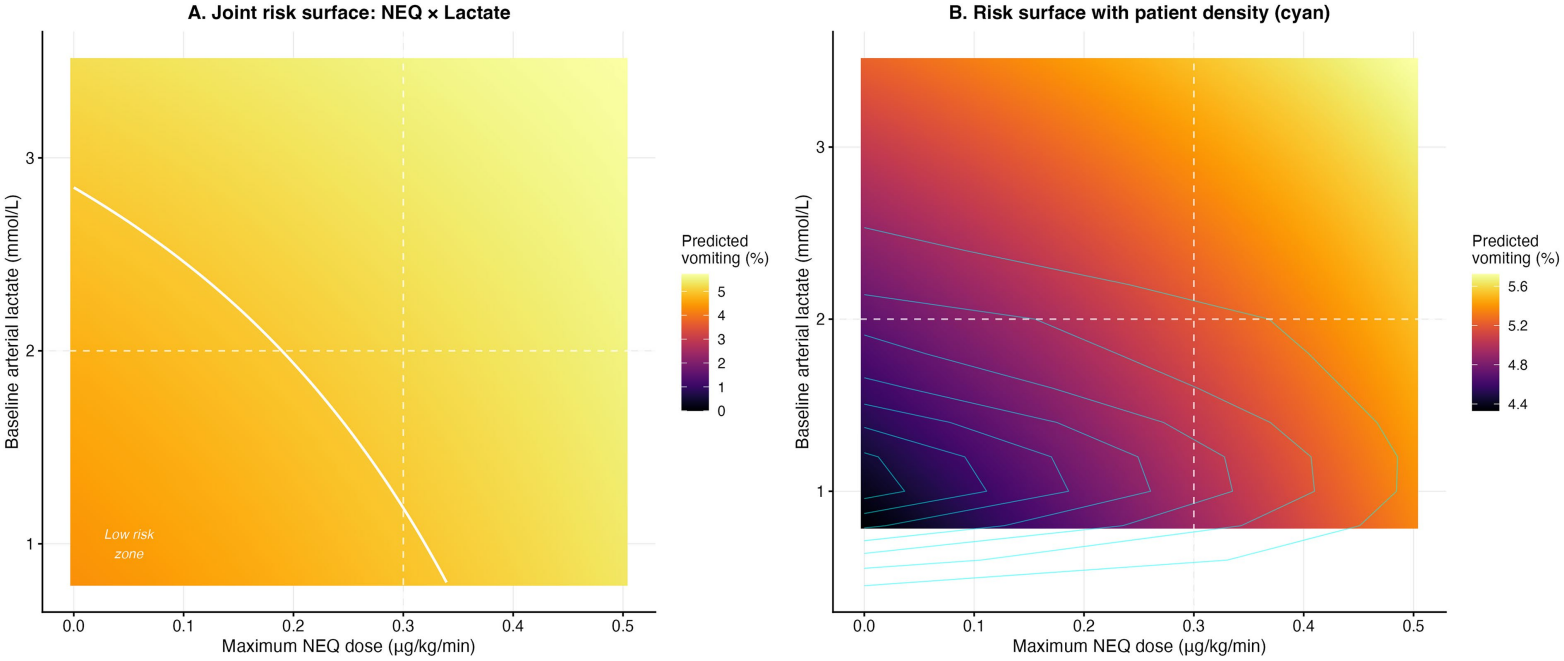
Joint NEQ × lactate risk surface from a generalized additive model with a tensor-product smooth. Panel **(A)**: predicted vomiting-risk surface, with contour lines indicating predicted probabilities. Panel **(B)**: the same risk surface overlaid with cyan patient-density contours. Color scales indicate predicted vomiting probability (%). Dashed reference lines mark NEQ 0.3 μg/kg/min and baseline arterial lactate 2 mmol/L.

The GAM joint smooth term did not reach significance (*p* = 0.11). The risk surface (Figure 2) showed a gradient from low-NEQ/low-lactate to high-NEQ/high-lactate, with highest predicted probabilities (≥8%) concentrated where NEQ > 0.3 and lactate >3 mmol/L. The risk gradient appeared steeper along the NEQ axis at higher lactate values, consistent with a modest synergistic pattern, though the overall surface fit remained modest, reflecting the multifactorial nature of EN intolerance.

Adding lactate did not materially improve discrimination (Supplementary Figure S4).

### Additive interaction analysis

On the additive scale, the doubly-exposed stratum (NEQ ≥ 0.3 with lactate >2 mmol/L) had an adjusted OR of 2.02 (95% CI 1.33–3.07, *p* = 0.001) versus reference. NEQ ≥ 0.3 was significantly associated with vomiting only among patients with elevated lactate (OR 1.87, *p* = 0.013), not among those with normal lactate (OR 1.36, *p* = 0.12).

On the additive scale, the relative excess risk due to interaction (RERI 0.58, 95% CI − 0.39 to 1.56), attributable proportion (AP 0.29, 95% CI − 0.12 to 0.70), and synergy index (2.34, 95% CI 0.45 to 12.12) all included the null value. The point estimates are directionally consistent with super-additivity, but the data do not provide statistical support for additive interaction, and these metrics should be interpreted as exploratory.

**Figure 3:**
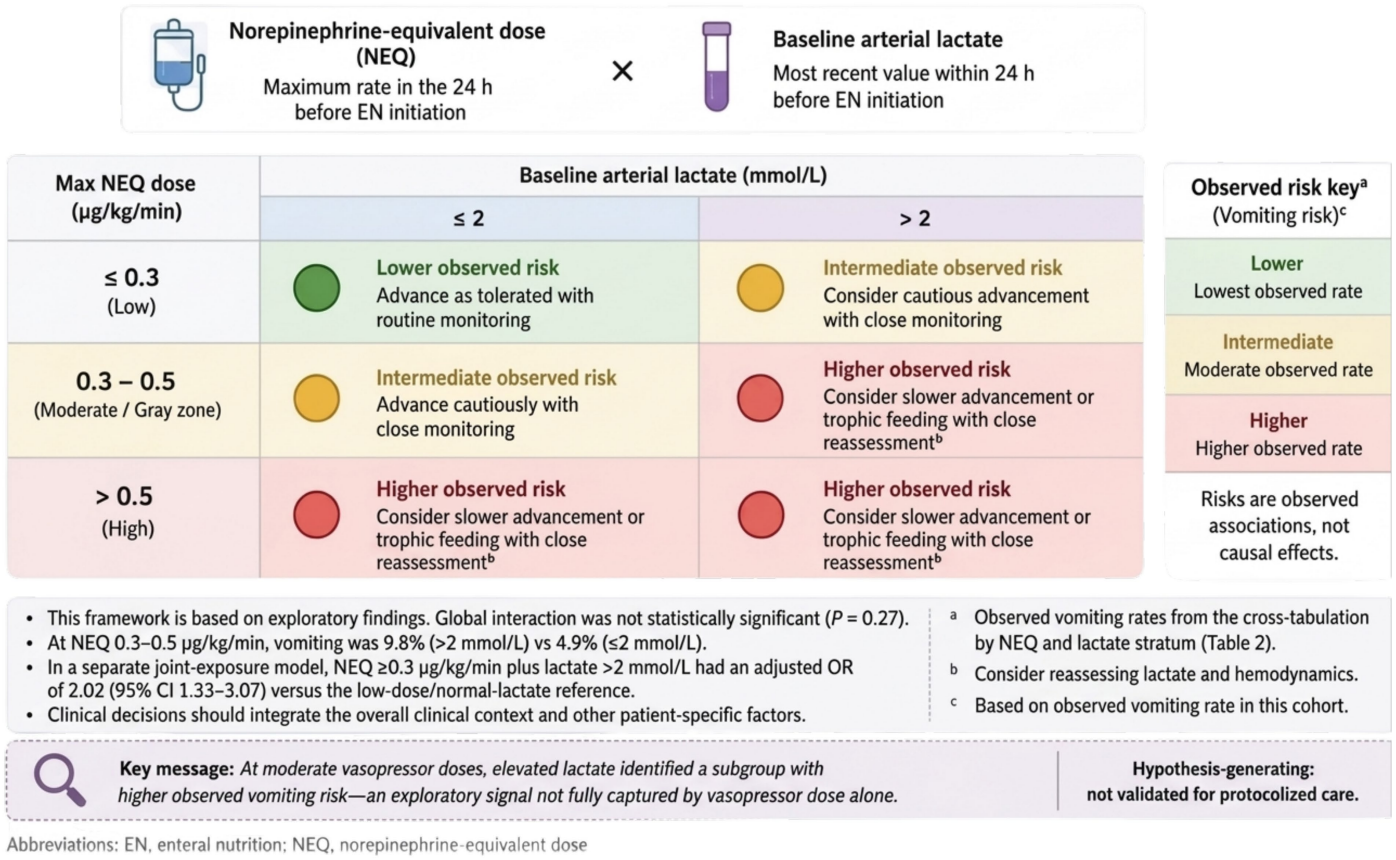
Exploratory risk-stratification framework relating norepinephrine-equivalent dose and baseline lactate to observed vomiting rates. This conceptual figure summarizes a non-significant, hypothesis-generating signal and is not a clinical practice recommendation; prospective validation is required. Created in BioRender. Li Y. (2026). https://BioRender.com/xdd1i82.

This exploratory subgroup signal is illustrated, for descriptive purposes only, in the candidate exploratory risk-stratification framework in Figure 3. The elevated point estimate in the doubly-exposed stratum reflects a localized association within the moderate-dose range that did not reach statistical significance on the global interaction test and requires prospective confirmation.

### Sensitivity analyses

All seven prespecified sensitivity analyses yielded consistent null interaction results (Supplementary Table S1; Supplementary Figure S2). A 6-h exposure window was borderline (*p* = 0.054), suggesting proximal vasopressor exposure may carry a stronger signal. Baseline characteristics of patients with and without available lactate were similar, supporting generalizability.

## Discussion

### Principal findings

Building on the NEQ–vomiting dose–response established in our companion study (5), this preregistered analysis examined whether arterial lactate identifies patients at differential risk within the moderate vasopressor dose range.

The principal finding was an exploratory subgroup signal: at NEQ 0.3–0.5 μg/kg/min, concurrent lactate elevation (>2 mmol/L) was associated with approximately doubled observed vomiting risk. A global multiplicative interaction was not detected (*p* = 0.27), consistent with this signal being localized to the moderate-dose range rather than operating uniformly across all vasopressor doses.

Several descriptive observations are directionally consistent with this signal, although none constitutes statistically significant effect modification: numerically steeper dose–response slopes in the elevated-lactate stratum (OR for NEQ 0 → 0.5: 1.53 vs. 1.08); an approximately doubled observed vomiting rate at NEQ 0.3–0.5 with elevated lactate (9.8% vs. 4.9%); a higher point estimate for the doubly-exposed stratum (adjusted OR 2.02, *p* = 0.001), with NEQ ≥ 0.3 reaching nominal significance only in the elevated-lactate group (*p* = 0.013 vs. *p* = 0.12); and a 6-h exposure-window interaction that only approached significance (*p* = 0.054). The low event rate (5.0%) and subgroup-concentrated signal likely limited formal detection power.

Effect modification versus confounding by severity. Because arterial lactate co-varies with both vasopressor dose and global illness severity, the observed stratum difference could reflect genuine effect modification or residual confounding by severity. Our interaction model conditions on dose (spline-modeled NEQ) and on non-cardiovascular SOFA, so the stratum-specific and interaction estimates are adjusted rather than marginal. Nevertheless, this observational design cannot fully separate a biological modifier from severity-driven confounding, because unmeasured determinants of severity—cardiac output, fluid balance, sedation depth, and the cardiovascular component of SOFA, which we intentionally omitted to avoid adjusting away the vasopressor exposure—may differ across lactate strata. We therefore interpret the moderate-dose signal as hypothesis-generating and explicitly not as evidence of an independent, dose-conditional effect modifier.

### Physiological rationale for lactate as an effect modifier

Biologically, the proposed lactate-related splanchnic susceptibility rests on the dual-hit model of splanchnic injury (9, 10). The splanchnic circulation is among the first vascular beds sacrificed during shock (11); when exogenous vasopressors are superimposed, mesenteric blood flow may fall below the threshold for gut mucosal integrity (12, 21).

NEQ and lactate capture overlapping but non-identical dimensions of hemodynamic compromise (22); lactate integrates global perfusion adequacy beyond pharmacological burden (8).

Animal data support this framework (23): enteral feeding during mesenteric hypoperfusion triggered mucosal injury not observed after perfusion normalization (24).

### Clinical implications

The concept of lactate-informed risk stratification has direct implications for bedside EN decisions. Current practice largely relies on vasopressor dose alone (3, 4); incorporating lactate may provide more nuanced risk stratification.

At NEQ 0.3–0.5, normal-lactate patients had a vomiting rate of 4.9% (comparable to baseline), whereas elevated-lactate patients had 9.8%. At lower doses (<0.2), lactate stratification did not meaningfully alter risk, implying that lactate adds the most value within the moderate-dose gray zone. Figure 3 illustrates these findings as an exploratory risk-stratification framework; observed vomiting rates are visualized in Supplementary Figure S3. This framework reflects a localized signal and should not be interpreted as evidence of a confirmed global interaction.

Practically, the joint NEQ × lactate assessment requires no additional testing beyond standard ICU monitoring (25).

The absence of improvement in global discrimination warrants explicit interpretation. The area under the ROC curve is a population-averaged summary dominated by the large, low-risk majority of patients. A modifier that acts only within a narrow exposure band—here, the moderate-dose stratum comprising roughly one-sixth of the cohort—and on an uncommon outcome (5%) will redistribute risk locally without materially shifting a global C-statistic. The unchanged AUC (base model 0.614 vs. lactate-expanded model 0.620; DeLong *p* = 0.25) is therefore consistent with, rather than contradictory to, a localized effect-modification signal, and implies that lactate is best framed as a within-stratum advancement-pacing aid rather than a stand-alone discriminative predictor.

Crucially, these findings are hypothesis-generating and should be read as a feed-advancement pacing signal rather than a withholding signal. Higher-risk NEQ × lactate combinations may warrant graduated monitoring: slower advancement, more frequent assessments, or earlier return to trophic rates (26).

These findings support a perfusion-informed strategy in which lactate calibrates advancement speed and monitoring frequency rather than denying EN. Specifically, lactate >2 mmol/L at NEQ 0.3–0.5 μg/kg/min may trigger slower rate escalation and more frequent tolerance checks in future protocolized studies.

### Relationship to the parent study

This analysis shares the same cohort, outcome definition, and covariates as the parent study (5), so that the only analytical addition is the lactate interaction term. The choice of vomiting as a standalone outcome—rather than a composite that includes lactate-related criteria—is methodologically essential: examining lactate as an effect modifier requires that it remain independent of the outcome definition.

### Limitations

First, vomiting as the sole outcome captured only overt intolerance; subtler forms such as high gastric residuals or abdominal distension were not assessed. Second, arterial lactate is non-specific—elevations may reflect hepatic dysfunction, aerobic glycolysis, or medication effects rather than splanchnic hypoperfusion (27). Moreover, lactate was clinically rather than protocolically timed, introducing potential measurement bias (28). Third, the study was powered for main effects; the interaction analysis may be underpowered, particularly in extreme subgroups, and the absence of significance does not exclude clinically relevant effect modification (29). Fourth, unmeasured confounders (sedation depth, prokinetic use, EN advancement rate, fluid balance, cardiac output) could not be adjusted for (5). Fifth, the single-center retrospective design limits generalizability; multicenter validation with serial lactate measurements and protocolized timing would strengthen both internal validity and transportability. Importantly, the observed effect modification appears localized to the moderate-dose range rather than uniformly distributed across all vasopressor exposures, consistent with the biological rationale that splanchnic vulnerability concentrates within this hemodynamic corridor.

## Conclusion

In this preregistered analysis of 5,542 critically ill patients, we observed an exploratory subgroup signal at moderate vasopressor doses (NEQ 0.3–0.5 μg/kg/min), where concurrent lactate >2 mmol/L was associated with an approximately doubled observed vomiting rate (9.8% vs. 4.9%). In a dichotomized joint-exposure analysis, a norepinephrine-equivalent dose ≥0.3 μg/kg/min combined with lactate >2 mmol/L was associated with an adjusted OR of 2.02 (95% CI 1.33–3.07) compared with the dose <0.3 μg/kg/min and lactate ≤2 mmol/L reference group. This association was concentrated in the moderate-dose range, precisely where clinical uncertainty about feeding advancement is greatest. Because the global interaction test was not statistically significant, these findings are hypothesis-generating: they identify a candidate lactate-stratified approach—slower advancement and closer monitoring at moderate doses with elevated lactate—that should be tested in prospective, protocolized studies before clinical application.

## Data Availability

Publicly available data were analyzed in this study. The MIMIC-IV v2.0 dataset used in this study is available through credentialed access at https://physionet.org/content/mimiciv/2.0/. The analytic SQL and R scripts are available on the Open Science Framework at https://osf.io/epjv6.

## Footnotes

1
https://osf.io/epjv6
