# Supplementary material for: Arterial lactate and enteral feeding intolerance within the vasopressor gray zone: a retrospective cohort study
Source: Front Nutr. 2026 Jul 24;13:1864125. doi: 10.3389/fnut.2026.1864125 (PMC13447207; doi:10.3389/fnut.2026.1864125)
Supplement: Supplementary file 1 [file Table_1.DOCX]

**Supplementary Materials**

Companion to: Arterial Lactate as a Bedside Signal to Refine Enteral Feeding Advancement Within the Vasopressor Gray Zone: A Retrospective Cohort Study

**Supplementary Materials**

Supplementary Table S1. Summary of sensitivity analyses for the lactate × NEQ interaction. All analyses tested the multiplicative interaction via likelihood ratio test. None reached conventional significance (P < 0.05), though the 6-hour exposure window analysis was borderline (P = 0.054).

| **Sensitivity analysis** | **N** | **Interaction P** |
| --- | --- | --- |
| S1a: Lactate >1.5 mmol/L threshold | 5,542 | 0.78 |
| S1b: Lactate >2.0 mmol/L (primary) | 5,542 | 0.27 |
| S1c: Lactate >4.0 mmol/L threshold | 5,542 | 0.46 |
| S2: Continuous lactate × RCS(NEQ) | 5,542 | 0.84 |
| S3: 6-hour pre-EN exposure window | 3,048 | 0.054 |
| S4: Mechanical ventilation adjustment | 5,542 | 0.27 |
| S5: MICU patients only | 2,613 | 0.31 |
| S6: 12-hour pre-EN exposure window | 3,577 | 0.11 |

Supplementary Figure S1. Heatmap of vomiting rates by NEQ dose category and lactate stratum. Cell values represent observed vomiting percentages. Color intensity reflects vomiting rate from low (light) to high (dark).

Supplementary Figure S2. Sensitivity analysis results. Bar chart showing −log10(P) for the interaction term across all prespecified sensitivity analyses. The dashed line indicates P = 0.05.

Supplementary Figure S3. Exploratory NEQ × lactate risk-stratification heatmap based on observed vomiting rates. Two-by-five heatmap of observed vomiting rates by NEQ dose category and lactate stratum (n = 5,542), with observed event counts annotated in each cell. The moderate-dose gray zone (NEQ 0.3–0.5 with lactate >2 mmol/L) is highlighted; observed vomiting rate 9.8% (20/204) versus 4.9% (17/347) in the normal-lactate counterpart cell. This figure is descriptive and hypothesis-generating and is not a clinical practice recommendation.

Supplementary Figure S4. Receiver operating characteristic curves comparing the base model (NEQ + covariates, AUC = 0.614) and the lactate-expanded model (NEQ × lactate interaction + covariates, AUC = 0.620). DeLong test P = 0.25. Net reclassification improvement (NRI = 0.008) and integrated discrimination improvement (IDI = 0.001) were both negligible.
